# Tissue Plasminogen Activator and MRI Signs of Cerebral Small Vessel Disease

**DOI:** 10.3390/brainsci9100266

**Published:** 2019-10-05

**Authors:** Larisa A. Dobrynina, Alla A. Shabalina, Maryam R. Zabitova, Elena I. Kremneva, Zukhra Sh. Gadzhieva, Marina V. Krotenkova, Elena V. Gnedovskaya, Alexander B. Berdalin, Lyudmila A. Kalashnikova

**Affiliations:** 1 Research Center of Neurology, 80 Volokolamskoe shosse, 125367 Moscow, Russia; ashabalina@yandex.ru (A.A.S.); m_zabitova@mail.ru (M.R.Z.); kremneva@neurology.ru (E.I.K.); zuhradoc@mail.ru (Z.S.G.); krotenkova_mrt@mail.ru (M.V.K.); gnedovskaya@mail.ru (E.V.G.); kalashnikovancn@yandex.ru (L.A.K.); 2 Federal State Budgetary Institution "Federal Center for Cerebrovascular Pathology and Stroke", 1, stroenie 10, Ostrovityanova, 117342 Moscow, Russia; alex_berdalin@mail.ru

**Keywords:** cerebral small vessel disease, white matter hyperintensities, perivascular spaces, endothelial dysfunction, tissue plasminogen activator, blood–brain barrier

## Abstract

Cerebral small vessel disease (SVD) is one of the leading causes of cognitive impairment and stroke. The importance of endothelial dysfunction and high blood–brain barrier (BBB) permeability in pathogenesis, together with ischemia, is under discussion. The aim of this study was to clarify the relationship between tissue plasminogen activator (t-PA), plasminogen activator inhibitor (PAI-1), and magnetic resonance imaging (MRI) signs of SVD. We examined 71 patients (23 men and 48 women; mean age: 60.5 ± 6.9 years) with clinical and MRI signs of SVD, and 21 healthy volunteers with normal MRIs. All subjects underwent 3T MRI and measurements of t-PA and PAI-1 levels. An increase in t-PA level is correlated with the volume of white matter hyperintensities (WMH) (*R* = 0.289, *p* = 0.034), severity on the Fazekas scale (*p* = 0.000), and with the size of subcortical (*p* = 0.002) and semiovale (*p* = 0.008) perivascular spaces. The PAI-1 level is not correlated with the t-PA level or MRI signs of SVD. The correlation between t-PA and the degree of WMH and perivascular spaces’ enlargement, without a correlation with PAI-1 and lacunes, is consistent with the importance of t-PA in BBB disruption and its role in causing brain damage in SVD.

## 1. Introduction

Cerebral small vessel disease (SVD) is associated with age and vascular risk factors, and is one of the leading causes of cognitive impairment, and ischemic and hemorrhagic strokes [1]. Given the technical limitations in visualizing small vessels, SVD is diagnosed in patients with clinical symptoms of SVD based on magnetic resonance imaging (MRI) signs of brain damage. These include white matter hyperintensities (WMH), recent small subcortical infarcts, lacunes, microbleeds, enlarged perivascular spaces, and atrophy [2]. WMH on MRI are defined as areas of hyperintense signal on T2-weighted sequences and iso- or hypointense signals on T1-weighted sequences [2]. Studies have established a link between the total SVD score on MRI and cognitive impairment, risk of recurrent stroke, and mortality after stroke [3,4,5]. Arterial hypertension (AH) is considered the main risk factor for the development of SVD, but no direct causal relationship exists in a significant number of cases [6,7]. The pathogenesis of SVD is not completely understood [8,9]. Endothelial damage has been shown to be mandatory for its development [10,11]. The role of endothelial dysfunction in coagulation and fibrinolytic disturbances, leading to lacunar infarcts, has been confirmed [12], whereas the results of studies concerning this mechanism in WMH, the other leading manifestation of SVD, are contradictory [8,13]. Researchers are considering the importance of the high permeability of the vascular wall and the overall state of the blood–brain barrier (BBB) in the development of WMH in association with endothelial dysfunction [8]. The study of endothelial activation parameters, which potentially affect both coagulation and fibrinolysis, and BBB permeability, is therefore particularly important in cerebral SVD research. Tissue plasminogen activator (t-PA), which is synthesized by endothelial cells, has long been known for its role in fibrinolysis [14], whereas its significance in altering BBB permeability has only been established more recently [15,16]. We hypothesized that an increase in t-PA production, associated with endothelial activation [17], can trigger and maintain the mechanisms of SVD development. A prospective study demonstrated the role of t-PA in SVD, with a correlation between t-PA activity and WMH and their progression found in patients with lacunar stroke [18,19]. Because WMH precede the formation of lacunes in a large proportion of patients, it is important to clarify the role of t-PA in the development of SVD, regardless of their presence, as well as in the formation of other MRI signs of SVD.

The aim of this study was to clarify the relationship between t-PA and plasminogen activator inhibitor (PAI-1) and MRI signs of cerebral SVD.

## 2. Materials and Methods

### 2.1. Participants

The study included patients aged 46–70 years with cognitive and other cerebral complaints, presenting to the Center of Neurology (Moscow, Russia) between January 2016 and December 2017, whose brain changes, as found by an MRI, were consistent with cerebral SVD (lacunes, WMH, enlarged perivascular spaces, microbleeds, and cerebral atrophy) [2]. Patients with stage I WMH on the Fazekas scale were included in the study if they had stage 2 or 3 hypertension and/or ≥1 lacunar infarction.

### 2.2. Exclusion Criteria

The exclusion criteria were as follows: (1) severe dementia; (2) cognitive impairment due to probable Alzheimer’s disease according to the U.S. National Institute on Aging criteria [20,21]; (3) patients with small subcortical infarcts/lacunes <3 months after an acute cerebrovascular event; (4) SVD due to other independent causes (genetic, inflammatory, thrombophilic, systemic, toxic or history of severe migraines); (5) other causes of stroke and concomitant brain pathology apart from SVD; (6) >50% atherosclerotic stenosis of extra- or intracranial arteries; (7) serious physical disease, including cardiac (ejection fraction <50%), endocrine (type 1 or type 2 diabetes mellitus with significant vascular complications or decompensated thyroid dysfunction), renal (chronic kidney disease if glomerular filtration rate <30 mL/min), or other; and (8) contraindications to MRI.

The control group consisted of volunteers with no clinical and neuroimaging evidence of cerebrovascular or degenerative pathology, matched in age and sex.

According to the above criteria, 71 patients (23 men and 48 women; mean age: 60.51 ± 6.76 years) and 21 volunteers (six men and 15 women; mean age: 57.33 ± 5.19 years) were included in the study.

The study was approved by the Local Ethics Committee of the Center of Neurology. All subjects signed an informed consent form, agreeing to the examination and processing of personal data.

All patients were asked about the appearance of general and neurological symptoms of the disease. Their physical status, major vascular risk factors [22], and neurological syndromes were assessed. The severity of cognitive impairment was determined concurrently using the Montreal Cognitive Assessment (MoCA) scale and independence in everyday life [23]. Dementia was diagnosed if the MoCA score was <26 points and the patient was dependent on others; cognitive impairment was considered mild if the score was <26 points and the patient was independent. Cognitive impairment was considered subjective if the score was ≥26 points with cognitive complaints.

Blood samples for the subsequent measurement of t-PA and PAI-1 levels were obtained in the morning by cubital fossa venipuncture, on an empty stomach, in a Vacuette-tube (Greiner bio-one, Kremsmünster, Austria) with a coagulation activator. The serum was separated from the blood cells by centrifugation for 10 min at 1500× *g* at room temperature. The resulting supernatant was transferred to separate, labeled microcentrifuge tubes and frozen at −80 °C. The samples were thawed at room temperature before being examined. Solid-phase sandwich enzyme immunoassay, eBioscience (Vienna, Austria) reagents and corresponding calibrators, and a Victor-2 microplate reader (Perkin Elmer, Waltham, MA, USA) was used. An ELISA assay was performed in duplicate using lyophilized control sera with low and high contents of the studied parameters.

MRI data were acquired using a Siemens MAGNETOM Verio 3T scanner (Siemens Medical Systems, Erlangen, Germany) with a standard 12-channel matrix head coil. To evaluate the STRIVE (Standards for Reporting Vascular changes on neuroimaging) criteria [2], patients and the control group underwent axial spin-echo T2-weighted imaging (TR 4000 ms; TE 118 ms; slice thickness 5.0 mm; in-plane resolution 1.5 mm^2^; duration: 2 min 02 s); sagittal three-dimensional (3D) T2 FLAIR (TR 6000 ms; TE 395 ms; isotropic voxel 1 × 1 × 1 mm; duration: 7 min 12 s); sagittal 3D T1-mpr (TR 1900 ms; TE 2.5 ms; isotropic voxel 1 × 1 × 1 mm; duration: 4 min 16 s); diffusion MRI (DWI) using axial spin-echo echo-planar imaging sequence with two b-values (0 and 1000 s/mm^2^) (TR 4000 ms; TE 100 ms; slice thickness 4 mm; duration: 1 min 20 s); axial susceptibility-weighted imaging (SWI) sequence with magnitude and phase images reconstruction (TR 28 ms; TE 20 ms; slice thickness 1.2 mm; FOV 179 × 230 mm^2^, duration: 8 min 12 s).

Two neuroradiologists (E.K. and M.V.) evaluated the brain MRI studies in a standardized manner and blinded to clinical information. No STRIVE criteria were found in the control group. For the patient group, no acute or recent small lacunar infarcts were found based on DWI analysis. White matter and basal ganglia lacunes were graded on T2 FLAIR images depending on their amount (<5, 5–10, and >10 lacunes). The Fazekas scale was used to quantify T2 FLAIR WMH (grades 0–3), as well as semi-automatic WMH segmentation using the LST toolbox for SPM8 [24], with further manual correction using the ITK-SNAP viewer [25]. Microbleed rating was performed on SWI images depending on their amount (<5, 5–10, and >10); microbleeds in basic ganglia, frontal, parietal, occipital, and temporary lobes were calculated separately. We used the data for the temporal lobe with the greatest severity for the comparisons. Perivascular spaces were graded based on their amount (<5, 5–10, and >10) and size (1–4 mm) in the centrum semiovale and basal ganglia. Most patients (98%) had more than 10 enlarged perivascular spaces in both areas, so further statistical analysis was performed only for their size.

Statistical analysis was performed using IBM SPSS 23.0 (IBM SPSS Statistics, version 23.0, IBM Corp., Armonk, NY, USA) and R 3.4.3 (R Foundation for Statistical Computing, Vienna, Austria) software. The main parameters for categorical and ordinal variables were frequency and proportion (%), and median and quartiles for quantitative variables. In all cases, two-way statistical criteria were used. The null hypothesis was rejected if *p* < 0.05.

Qualitative parameters were compared using the χ^2^ test or Fisher’s exact test, according to the grouping variable levels.

Quantitative parameters were compared using the Kruskal–Wallis test with subsequent pairwise comparisons using the Mann–Whitney *U* test with Bonferroni correction. Spearman’s rank correlation was used to assess the relationship between the volume of WMH and t-PA and PAI-1 levels, as well as the relationship between these two blood parameters.

## 3. Results

The main demographic data and risk factors in the examined patients and the control group subjects are presented in Table 1. Both the group with SVD and the control group were predominantly female. The presence and degree of hypertension and diabetes mellitus differed significantly between the patients with SVD and the control group.

Clinical symptoms of SVD are presented in Table 2. The leading symptoms were cognitive impairment of varying severity and gait disorders unrelated to hemiparesis.

**Figure 1:**
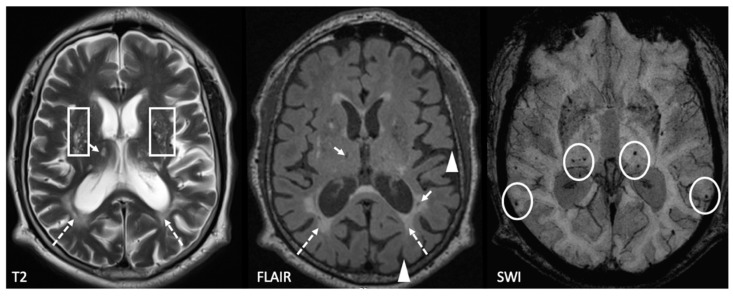
Example of characteristic SVD MRI features: dotted arrows—white matter hyperintensities (WMH), solid arrows—lacunes, circles—microbleeds, triangles—sulcus enlargement as a marker of brain atrophy, and rectangles—multiple enlarged perivascular spaces.

The main neuroimaging features of SVD are demonstrated in Figure 1. The type and degree of MRI signs of SVD and their comparative analysis with t-PA and PAI-1 levels are presented in Table 3. The t-PA level showed a significant correlation with the degree of WMH and the size of perivascular spaces, both in the centrum semiovale and the basal ganglia (Table 3). No other significant correlations between t-PA and MRI signs were identified. The PAI-1 level was not correlated with the t-PA level or MRI signs of SVD.

**Figure 2:**
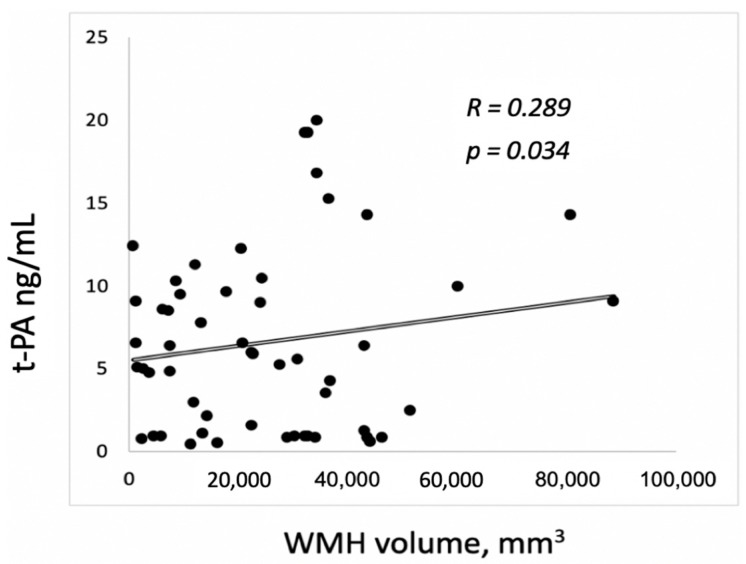
The relationship between t-PA level and WMH volume.

A direct relationship between the level of t-PA and degree of WMH was confirmed by comparing t-PA to the total volume of WMH (*R* = 0.289, *p* = 0.034; Figure 2), but this was not evident for PAI-1.

## 4. Discussion

In this study, we established a link between the t-PA level and the volume and severity of WMH on the Fazekas scale, as well as the size of perivascular spaces. The patterns identified in the signs of SVD associated with increased BBB permeability [26,27] indicate the involvement of t-PA in this process. Although patients with SVD differed from the volunteers in terms of their degree of hypertension, we found no evidence that hypertension affected the level of t-PA. This suggests that t-PA’s importance in SVD is independent of hypertension. The role of t-PA in the formation of WMH was previously established in a prospective study: an association between t-PA activity and WMH and their progression was found in patients with lacunar stroke [18,19]. Our study showed that t-PA has an independent effect on the severity of WMH, unrelated to lacunes. Notably, we established t-PA’s role in the formation of enlarged perivascular spaces for the first time. Enlarged perivascular spaces have been included as an independent MRI sign in SVD study standards (STRIVE) [2] because they have been recognized as markers of high BBB permeability and immune-mediated brain damage [8]. The established correlations shed a different light on the role of endothelial t-PA in small vessel damage. t-PA belongs to the family of serine proteases. Its ability to convert plasminogen into plasmin by breaking down fibrin clots is well known. The serum t-PA inhibitor is PAI-1, whereas neuroserpin is the selective inhibitor in the central nervous system. Researchers have only recently become interested in the nonfibrinolytic properties of t-PA [28]. The direct t-PA dose-dependent increase in BBB permeability and the effect of t-PA on the morphology of endothelial cells and astrocytes through the Rho-kinase pathway were shown in in vitro models of the BBB [29]. Among the mechanisms of t-PA-mediated disruption of BBB permeability, the degradation of extracellular matrix proteins by the direct action of plasmin and through the activation of matrix metalloproteinases, in particular MMP-3, is currently being discussed [30]. The typical morphological phenomenon in SVD—acute fibrinoid necrosis in perforating artery walls with increased endothelial permeability—has previously been established by introducing plasmin into murine brains [31]. The role of t-PA in increasing BBB permeability in traumatic brain injury [32] and ischemic stroke is being actively studied [33]. t-PA participates in neurovascular coupling, microglial activation and inflammation, neuronal plasticity, and many other processes [34]. 

## 5. Conclusions

The identified correlation between t-PA level, WMH severity, and the size of perivascular spaces in patients with SVD—MRI signs of SVD associated with endothelial dysfunction, increased BBB permeability, and immune-mediated brain damage—correspond to the previously, experimentally established participation of t-PA in these pathological processes and their importance in the progress of SVD and its clinical manifestations. Further study of the parameters pathogenetically associated with t-PA and the development of SVD will expand our understanding of the mechanisms of small vessel and cerebral damage, with the ultimate goal of developing pathogenetically justified therapeutic approaches.

## Figures and Tables

**Table 1:** Main demographic parameters and risk factors in patients with cerebral small vessel disease (SVD) and in the control group.

Parameter	SVD (*n*, %)	Control (*n*, %)	*p*
Age (years)	60.51 (±6.76)	57.33 (±5.19)	0.792
Sex			
Female	48 (67.6%)	15 (71.4%)	0.74
Arterial hypertension (AH)	59 (83.1%)	9 (42.86%)	0.0002
Degree of AH			
1	7 (11.86%)	5 (55.55%)	0.002
2	15 (25.42%)	3 (33.33%)	
3	37 (62.71%)	1 (11.11%)	
Type 2 diabetes mellitus	15 (21.13%)	0 (0%)	0.02
Smoking	19 (26.76%)	9 (42.86%)	0.12
Body mass index	34.02	26.95	0.165

**Table 2:** Clinical symptoms in patients with SVD.

Clinical Symptom	SVD, *n* (%)
Cognitive impairment	
Dementia	12 (16.9%)
Mild cognitive impairment	30 (42.25%)
Subjective cognitive impairment	29 (40.84%)
Gait disorders unrelated to hemiparesis	40 (56.3%)
Hemiparesis	3 (4.2%)
Pseudobulbar syndrome	5 (7.04%)
Urinary disturbances	
Urinary frequency	16 (22.5%)
Urinary incontinence	13 (18.3%)

Note: The levels of t-PA and PAI-1 were independent of the presence and degree of hypertension (*p* = 0.140 and *p* = 0.643, respectively).

**Table 3:** Comparative analysis of the levels of tissue plasminogen activator (t-PA) and plasminogen activator inhibitor (PAI-1) with MRI signs of SVD.

MRI Signs of SVD	*n*	t-PA (ng/mL) Me (Q25%; Q75%)	*p*	PAI-1 (ng/mL) Me (Q25%; Q75%)	*p*
**WMH**			**0.000**		0.150
F1	17	1.0 (0.91; 5.6)		26.0 (23.4; 34.2)	
F2	24	4.9 (1.45; 8.8)		20.8 (15.8; 27.2)	
F3	30	9.05 (5.9; 14.30)		26.3 (17.8; 36.6)	
**Lacunes** Basal ganglia					
None	45	4.7 (0.96; 8.5)	0.069	23.5 (16.8; 34.2)	0.442
<5	10	7.5 (5.0; 11.8)		26.4 (19.0; 35.2)	
5–10	6	10.8 (8.8; 15.0)		28.6 (19.3; 39.5)	
>10	10	8.85 (4.9; 12.3)		28.2 (19.2; 9.4)	
**Cerebral white matter**					
None	39	3.6 (0.95; 8.6)	0.051	23.4 (16.8; 32.3)	0.175
<5	12	7.15 (5.05; 9.3)		26.0 (22.8; 37.8)	
5–10	7	9.10 (4.9; 14.3)		19.2 (12.7; 27.5)	
>10	13	10.3 (6.0; 11.8)		35.2 (19.3; 39.4)	
**Microbleeds** Basal ganglia					
None	48	5.0 (0.9; 8.7)	0.071	25.2 (19.4; 34.7)	0.917
<5	10	8.8 (0.98; 15.0)		19.9 (12.7; 38.8)	
5–10	5	9.50 (8.6; 10.5)		25.8 (19.0; 35.8)	
>10	8	11.3 (5.7; 14.8)		23.0 (16.5; 34.2)	
Temporal lobes					
No	53	5.0 (0.98; 8.8)	0.160	25.1 (19.3; 5.2)	0.600
<5	9	10.3 (9.1; 11.8)		19.2 (13.9; 26.7)	
5–10	2	7.42 (0.54; 14.30)		25.8 (16.4; 35.2)	
>10	7	8.6 (5.3; 15.3)		30.5 (19.0; 37.9)	
**Perivascular spaces** Basal ganglia					
3 mm	12	9.75 (6.15; 12.05)	**0.002**	23.1 (17.1; 37.9)	0.657
4 mm (single)	6	10.10 (8.6 ± 19.3)		17.0 (12.7; 30.5)	
Centrum semiovale					
2 mm	26	8.85 (0.92; 20.0)	**0.008**	27.2 (19.2; 35.8)	0.791
3 mm	3	9.7 (0.92; 20.0)		19.5 (13.9; 53.5)	

Note: In 70% of patients, multiple microbleeds (>10) were found both in the white matter of the temporal lobe and the basal ganglia. Bold style notes significant correlation.
